# Use of alcohol and drugs among health professionals in Norway: a study using data from questionnaires and samples of oral fluid

**DOI:** 10.1186/1745-6673-9-8

**Published:** 2014-03-10

**Authors:** Hilde Marie Erøy Edvardsen, Ritva Karinen, Inger Synnøve Moan, Elisabeth Leere Øiestad, Asbjørg Solberg Christophersen, Hallvard Gjerde

**Affiliations:** 1 Division of Forensic Sciences, Norwegian Institute of Public Health, P.O. Box 4404, Nydalen, N-0403 Oslo, Norway; 2 Norwegian Institute for Alcohol and Drug Research, P.O. Box 565, Sentrum, N-0105 Oslo, Norway

**Keywords:** Ethanol, Illicit drugs, Medications, Prevalence, Psychoactive substances, Saliva, Self-report, Workplace drug testing

## Abstract

Working under the influence of drugs and/or alcohol may affect safety and job performance. However, the size of this possible problem among health professionals (HPs) is unknown. The aim of this study was threefold: (i) to analyze samples of oral fluid and self-reported data from questionnaires to investigate the prevalence of alcohol and drugs among a sample of HPs in Norway, (ii) to study self-reported absence from or impairment at work due to alcohol and/or drug use, and (iii) to examine whether such use and absence/impairment due to such use depend on socio-demographic variables.

A total of 916 of the 933 invited HPs from hospitals and pharmacies participated in the study (participation rate = 98.2%), and 81.1% were women. Associations were analyzed in bi-variate cross tables with Chi-square statistics to assess statistical significance.

Alcohol was not detected in any of the samples. Ethyl glucuronide, a specific alcohol metabolite, was found in 0.3% of the collected samples. Illicit drugs and medicinal drugs were identified in 0.6% and 7.3% of the samples, respectively. Both analytical results and self-reported use of alcohol and drugs during the past 12 months indicate that recent and past year alcohol and drug use was lower among HPs than among workers in other business areas in Norway, Europe and US. Nevertheless, several HPs reported absence from work due to alcohol (0.9%) and medicinal drug use (0.8%) during the past 12 months. A substantial part (16.7%) of the self-reported medicinal drug users reported absence from work because of use of medicinal drugs during the past 12 months, and more than 1/4 of those reported in-efficiency at work because of the use of medicinal drugs during the past 12 months. Reduced efficiency at work due to alcohol use during the past 12 months was reported by 12.2%.

This sample of HPs seldom used illicit drugs, few had a high level of alcohol consumption, and few tested positive for medicinal drugs. Absence or hangover related to the use of medicinal drugs or alcohol appeared to be a bigger issue than the acute intoxication or the use of illicit drugs.

## Background

Alcohol and drug use may both have acute and long term consequences for the workplace. It is well known that such use and/or hangover effects may cause an increased risk for accidents, faults, inefficiency, and absence from work [1-4], all undesired effects.

Health professionals (HPs) are employees that have easy access to medicines at work, and the consequences of abuse may affect both themselves and patients [5]. The psychoactive effects of drugs may be caused by an acute pharmacological effect or hangover effect that influences the performance of the user even though the active compounds are cleared from the brain. Therefore, the effects of drugs and alcohol may reduce the effectiveness at work for many hours after drug intake. In addition, dependence may cause a sense of craving which may result in HPs being unfocused at work [5].

The Norwegian Board of Health Supervision withdraws the authorization from several HPs every year due to impairment by psychoactive compounds at work [6]. The true extent of HPs being unfit to work due to impairment is not known, and surveys aimed at estimating the prevalence have not previously been conducted.

Workplace Drug Testing (WDT) is one method to investigate the prevalence of drug use among employees. It is performed by analyzing biological samples, primarily urine, but other matrices such as oral fluid (OF) and hair may also be used for that purpose. In Norway, WDT is not as common as in Sweden or in some other European countries [7], and it is mainly done within the transport, petrochemical, shipping, automobile, pharmaceutical and computer industries, and urine is the matrix of choice. In the US, workplace testing is more widespread and also mandatory for many lines of businesses [8,9], the prevalence of positive urine samples has decreased from 13.6% in 1988 to 3.6% in 2009/2010 [8].

Studies on alcohol and drug use, and the extent of working under the influence of those types of substances may be performed by collecting self-reported information in addition to analysis of biological samples, which provides information about recent alcohol and drug use. The length of time the sample will be positive after substance use depends on type of matrix, taken dose, time between substance use and sampling, and analytical cut-off value. In addition, the number of substances in the panel is important; it should cover the most commonly used and misused drugs. Specific and sensitive analytical methods will not detect other compounds than those that are included in the test panel. A positive finding in OF typically indicates drug use during the past 24–48 hours [10]. A negative finding does not prove non-use of a substance; it merely indicates that the probability of recent use of the substances in the test panel is very small. By using questionnaires, one can obtain a wider picture (compared to biological samples taken once) of the pattern of alcohol and drug use, e.g., how frequently a person typically drinks during a 12 month period, how frequently a person drinks to intoxication and/or experiences amnesia and/or other unpleasant effects due to alcohol use during the same period. Moreover, by using questionnaires it is also possible to get more information about the consequences alcohol and drug use may have for the workplace, e.g., in terms of sickness absence, reduced productivity, etc. [4,11]. Finally, this information can be used to determine the strength of the relationship between alcohol and drug use and the consequences of such use for the workplace. One drawback with self-reported data is the risk of under-reporting, but over-reporting may also occur.

The prevalence of alcohol and drug use among employees in Norway has received scant research attention. One previous Norwegian study on alcohol habits among medical doctors have been conducted [12]. Postal questionnaires were collected, and the response rate was 86%. The study revealed that surgeons (and women in particular) drank more frequently and more hazardously than other doctors [12]. However, other HPs were not included and the study did not measure the extent of the employees being unfit to work due to impairment. Another previously conducted study among employees in the private sector in Norway was also based on questionnaires only to estimate the use of alcohol and drugs during the past 12 months [13]. A few other business areas in Norway have been evaluated in smaller studies [11,14], but WDT with biological samples among HPs has not yet been undertaken. One Norwegian pilot study was conducted in 2008–2009, and analyses of OF and self-reported information were combined to estimate alcohol and drug use among Norwegian employees [11]. This study did not include HPs.

From studies conducted in the general population in Norway, we know that young people have a higher alcohol consumption and that they more frequently used illicit drugs than older people, that men both drink alcohol and use illicit drugs more frequently than women, whereas medicinal drugs are more frequently used by older than younger individuals and more often by women than men [15-17]. Finally, people with a higher level of education drink more frequently but less alcohol on each drinking occasion than those with a lower level of education [15]. Age and gender differences may vary across different fields of work, e.g. as demonstrated by Rosta and Aasland [12].

### Aims of the study

In light of the different qualities of the two methodological approaches described above, the first aim of this study was to estimate alcohol and drug use among a selection of HPs in Norway using analysis of OF and self-reported data from questionnaires. OF was chosen for its easy sampling compared to blood and urine. The participation rate is higher when collecting OF compared to urine [18]. The second aim was to study self-reported absence from or impairment/hangover work due to alcohol or drug use during the past 12 months. Finally, in light of the findings from previous studies conducted in the general population showing that alcohol and drug use vary significantly according to age, gender and educational level [15-17], the third aim of this study was to examine whether the prevalence of alcohol and drug use among this group of HPs partly depends on these socio-demographic variables.

## Methods

The study was performed by using samples of OF and self-reported data on alcohol and drug use by means of questionnaires. The study was conducted from December 2010 to May 2012. A randomized selection of employees at 3 hospitals and all workers at 4 hospital pharmacies were asked to participate. The hospital workers were included if their clinic/department were randomly selected to participate, and all workers (nurses, medical doctors, paramedics, secretaries, physiotherapists, cleaning personnel etc.) at the chosen clinics/departments were included to provide workers with different background and educational attainment. The pharmacies provided pharmacists, pharmacy technicians, secretaries, nurses, etc.

The participants did not receive any remuneration. The project team members did however actively encourage the participation. The employers supported the study, and thorough planning and on-site arrangement was provided.

## Materials

The participants reported age group, gender, level of education, and answered questions about alcohol abstinence (12 month), recent (24 hour) alcohol use and medicinal and illicit drug use (48 hours and 12 months), in-efficiency or hangover at work- and absence from work due to alcohol or drug use.

Other variables were the presence of alcohol, medicinal and illicit drugs in samples of OF (see Table 1).

**Table 1:** The analytes and their LLOQ values, with prevalence in per cent of all tests and in per cent of all positive tests (n = 907)

**Compound**	**Description**	**LLOQ value**^**d **^**(ng/mL)**	**Prevalence of all tests (%)**	**Prevalence of all positive tests (%)**
6-Acetylmorphine (6-AM)	Metabolite of heroin	0.46	0	0
Alcohol		0.060 g/L	0	0
Alprazolam	Benzodiazepine^e^; anxiolytic	0.19	0	0
7-Aminoclonazepam (7-AC)	Metabolite of clonazepam^e^	0.17	0	0
7-Aminoflunitrazepam (7-AF)	Metabolite of flunitrazepam^e^	0.060	0	0
7-Aminonitrazepam (7-AN)	Metabolite of nitrazepam^e^	0.15	0	0
Amphetamine	Stimulant^c^	1.5	0	0
Benzoylecgonine	Metabolite of cocaine	3.3	0.11	1.4
Buprenorphine	Opioid^e^ used as analgesia and for opioid dependence	0.94	0.11	1.4
Clonazepam	Benzodiazepine^c,e^; anticonvulsant, anxiolytic	0.19	0	0
Cocaine	Stimulant^b^	0.42	0.22	2.7
Codeine	Opioid analgesic^e^, antitussive	0.60	0.55	6.8
Diazepam	Benzodiazepine^e^; anxiolytic, anticonvulsant, sedative	0.11	1.2	4.1
Ethyl glucuronide (EtG)	Metabolite of alcohol	5.7	0.3	4.1
Fenazepam	Benzodiazepine^c,e^; anxiolytic^a^	0.21	0	0
Fentanyl	Opioid analgesic^e^	0.20	0	0
Flunitrazepam	Benzodiazepine^e^; anxiolytic^a^	0.060	0	0
3,4-Methylenedioxy-methamphetamine (MDMA)	Illegal psychedelic hallucinogenic drug	8.6	0	0
Methadone	Opioid^e^ used mainly for opioid dependence, but also for analgesia	3.5	0	0
Methamphetamine	Stimulant^b^	1.7	0.11	1.4
Morphine	Opioid analgesic^e^, also metabolite of codeine and heroin	1.3	0.22	2.7
Nitrazepam	Benzodiazepine^e^; anxiolytic	0.17	0	0
Nordiazepam	Metabolite of diazepam^e^	0.11	1.1	14
Oxazepam	Benzodiazepine^e^; anxiolytic, anticonvulsant, and metabolite of diazepam	0.17	1.3	16
Oxycodone	Opioid analgesic^e^	2.1	0	0
Δ9-Tetrahydrocannabinol (THC)	Cannabis^c^	0.63	0.11	1.4
Tramadol	Opioid analgesic^e^	8.8	0.11	1.4
Zolpidem	Short acting z-hypnotic^e^	0.43	0.33	4.1
Zopiclone	Short acting z-hypnotic^e^	0.54	4.0	50
Benzodiazepines			3.1	37.8
Illicit drugs			0.6	6.8
Opioids			1.0	12.2
Medicinal drugs			7.3	89.2
Z-hypnotics			4.4	54.1
Total positive of all workers			8.2	100

*Note:*^a^Not marketed in Norway, ^b^Illegal in Norway, ^c^Mostly used illegally in Norway, ^d^Concentrations in neat OF, ^e^Classified as a medicinal drug.

### Ethics

The dataset was completely anonymous, and the study was approved by the Regional Committee for Medical and Health Research Ethics, Norway.

### Consent

Oral informed consent was obtained from the participants for publication of reports.

### Study design and setting

The recruitment of employees was done unannounced the upcoming quarter after the workers had received thorough information about the study. Representatives from the Norwegian Institute of Public Health (NIPH) visited each workplace and handed out envelopes containing an information leaflet about the study, a sampling kit for OF (StatSure Saliva Sampler^TM^, Statsure Diagnostic Systems, Framingham, MA) and the questionnaire to each employee that they met the actual day. Statsure Saliva Sampler^TM^ was chosen because it enabled the analysis of ethyl glucuronide (EtG) [19] and because the samples are stable for more than 24 hours before they need to be frozen after collection [20]. This is very useful when travelling is necessary for collection. The participants were given oral and written information about the study and a detailed instruction on how to collect OF in the correct manner. Staff from NIPH was always available to instruct and help the participants. All the employees had the opportunity to participate and provide data in privacy, which made the participation (or no participation) anonymous to colleagues and the study team. The time used for sample collection and answering questions was approximately five minutes. Questionnaires and OF samples were placed in the same, unlabeled, envelope, which was sealed and collected by the study team within a few hours. The data collection was performed during weekdays only.

### Sample preparation and analysis

The samples of OF were frozen shortly after collection and thawed once before analysis. All compounds analyzed, with their lower limit of quantification (LLOQ), are presented in Table 1.

Alcohol was analyzed by an automated enzymatic method using alcohol dehydrogenase [21]. EtG was analyzed using UPLC-MS/MS (Acquity UPLC and Quattro Premier XE, Waters Corporation, Milford, MA, USA) with a HSS T3 (1.8 μm 2.1 × 100 mm) column with a gradient elution of the mobile phase with methanol and 0.1% acetic acid after solid phase extraction (Hyper-SEP SAX/3 ml/200 mg, Thermo Fisher Scientific Inc., Boston, MA,USA) [19]. The other compounds (Table 1) were isolated by liquid-liquid extraction using ethyl acetate/heptane 4/1 (v/v) after addition of 0.2 M ammonium carbonate buffer pH 9.3 and analyzed using UPLC-MS/MS (Acquity UPLC and Quattro Premier XE, Waters Corporation, Milford, MA, USA) with a BEH C18 (1.7 μm 2.1 × 50 mm) column with a gradient elution of the mobile phase with methanol and 5 mM ammonium bicarbonate buffer pH 8.5 [22]. Concentrations in neat OF were calculated assuming that 1 mL OF was collected and diluted with 1 mL buffer in the sampling device.

### Statistical methods

Statistical analyses were carried out using PASW® Statistics (SPSS version 20) and Microsoft Excel 2010. Associations were analyzed in bi-variate cross tables. Chi-square statistics were used to assess statistical significance. The level of statistical significance was set as *p < 0.05.*

## Results

### Participants

A total of 933 HPs were invited to participate, representing hospital departments (n = 778) and hospital pharmacies (n = 155) (Tables 2 and 3). A total of 916 employees participated (participation rate 98.2%); 916 completed the questionnaire, and 907 samples of OF were collected. The study population consisted of HPs from 7 different workplaces, and their background and education were quite wide, giving a variety in this population. The distribution of gender, age, workplace and educational level for the participants was evenly distributed and is presented in Tables 2 and 3. The exception from the even distribution was, however, that nearly half of the participants from the hospital pharmacies had education at the upper secondary school level (49%), whereas the majority of the hospital workers had college/academy/university education (82%).

**Table 2:** Sample characteristics and prevalence of positive oral fluid samples by gender, age, workplace and educational level

	**Proportion (%)**	**Positive sample (%)**	**Medicinal drugs (%)**^**b**^	**Illicit drugs (%)**	**Benzodiazepines (%)**	**Z-hypnotics (%)**	**Opioids (%)**
Gender^a^ (n = 899)		^NS^	^NS^	^NS^	^NS^	^NS^	^NS^
Men	18.9	7.1	6.5	0.6	4.8	3	1.8
Women	81.1	8.5	7.5	0.6	2.6	4.9	0.8
Age group^a^ (n = 903)		^NS^	^NS^	^NS^	^NS^	p = 0.031	^NS^
<30	15.1	6.7	6.7	0	4.5	1.5	1.5
30-39	27.1	4.5	4.1	0	1.7	2.5	0.4
40-49	27.1	9.1	7.9	1.2	2.5	5.8	1.2
50-59	22.4	10.9	9.5	0.5	5	5.5	0.5
60+	8.3	13.3	12	1.3	2.7	9.3	2.7
Age 40^a^ (n = 903)		p = 0.006	p = 0.023	p = 0.056	^NS^	p = 0.004	^NS^
<40	42.2	5.3	5.1	0	2.7	2.1	0.8
40+	57.8	10.4	9.1	1	3.5	6.2	1.2
Workplace (n = 933)		p = 0.019	p = 0.018	^NS^	^NS^	^NS^	^NS^
Pharmacy	16.6	3.4	2.7	0.7	0.7	2	0
Hospital	83.4	9.1	8.2	0.5	3.6	4.9	1.2
Educational level^a^ (n = 852)		^NS^	p = 0.014	^NS^	^NS^	^NS^	^NS^
Primary and secondary school	2.1	16.7	16.7	0	5.6	11.1	0
Upper secondary school	21.1	4.6	2.9	1.1	1.1	1.7	0
College/academy/university	76.8	9.2	8.5	0.5	3.5	5.1	1.2

^a^Age, gender and educational level were unknown for some participants.

^b^The medicinal drugs includes opioids that may be prescribed.

^NS^no significant association.

**Table 3:** Self-reported absence and in-efficiency at work due to alcohol use during the past 12 months, medicinal drug use during the past 48 hours and illicit drug use during the past 12 months by gender, age, workplace and educational level

	**Proportion (%)**	**Absence due to alcohol (%)**	**In-efficiency/Hangover due to alcohol (%)**	**Medicinal drug use (%)**	**Illicit drug use (%)**
Gender^a^ (n = 899)		^NS^	^NS^	^NS^	^NS^
Men	18.9	1.8	14.7	6.0	10.6
Women	81.1	0.7	11.8	4.4	6
Age group^a^ (n = 903)		^NS^	p < 0.001	p = 0.015	p < 0.001
<30	15.1	1.5	22.1	1.5	24.2
30-39	27.1	0.8	15.5	2.0	8.6
40-49	27.1	1.2	10.2	6.7	3.1
50-59	22.4	0.5	6.9	6.5	1.6
60+	8.3	0	4.0	8.1	0
Age 40^a^ (n = 903)		^NS^	p < 0.001	p = 0.001	p < 0.001
<40	42.2	1	17.8	1.9	13.6
40+	57.8	0.8	8.0	6.8	2.1
Workplace (n = 933)		^NS^	^NS^	p = 0.034	^-^
Pharmacy	16.6	1.9	12.3	1.3	0
Hospital	83.4	0.7	12.2	5.3	6.8
Educational level^a^ (n = 852)		^NS^	p = 0.002	p = 0.028	^NS^
Primary and secondary school	2.1	0	5.6	16.7	0
Upper secondary school	21.1	0.6	5.0	2.8	5.9
College/academy/university	76.8	1.1	14.4	4.9	7.1

^a^Age, gender and educational level were unknown for some participants.

^NS^no significant association.

### Analysis of oral fluid

As presented in Table 1, 8.2% of the OF samples contained at least one compound, and some of the compounds analyzed for were not detected. EtG is a specific biomarker of alcohol intake that can be analyzed in biological samples. The prevalence was very low as shown in Table 1. Illicit drugs were found in 0.6% of the samples. The most frequently detected illicit drug was cocaine (0.3%). No employees below the age of forty tested positive for illicit drugs in this study (Table 2). The prevalence of medicinal drugs including opioids, benzodiazepines and z-hypnotics is presented in Table 1. Z-hypnotics were most frequently detected (4.3%). The second largest medicinal drug group found was the benzodiazepines, which were found in 3.1% of the samples. The opioids were the smallest group of medicinal drugs that was detected. A total of 1% tested positive for an opioid.

No significant gender differences were found in the prevalence of medicinal drugs in OF, but it was significantly higher among those aged above 40 years (χ^2^ (1, N = 893) = 5.185, p = 0.023). Moreover, the prevalence of medicinal drug use was significantly higher among employees in hospitals than among pharmacy workers (χ^2^ (1, N = 907) = 5.572, p = 0.018). Finally, medicinal drugs were more frequently used by those with primary and secondary school as their highest educational level than by those with a higher educational level (χ^2^ (2, N = 842) = 8.525, p = 0.014).

### Results from questionnaires

Some of the questionnaires were incompletely filled in. Therefore gender, age group and educational level are not known for some cases. The principal results of the questionnaire are presented in Table 3.

Thirteen per cent of the respondents had consumed alcohol during the past 24 hours, and 5.7% of them had consumed four or more drinks during the past 24 hours. About 1% of the respondents had been absent from work due to drinking alcohol during the past 12 months, and 12.2% had experienced in-efficiency or hangover at work due to alcohol use during the past 12 months. Nearly 13% consumed six drinks or more during one drinking session at least once a month during the past 12 months. Finally, 9.1% of the employees reported not drinking any alcohol during the past 12 months.

The prevalence of reporting absence from work due to drinking alcohol during past 12 months was fairly similar across genders, age groups, the various workplaces and among persons with different educational levels (Table 3). Self-reported in-efficiency or hangover at work due to alcohol during the past 12 months was significantly lower in the age group above 40 years (χ^2^ (1, N = 903) = 19.780, p = 0.000) and significantly higher with increasing educational level (χ^2^ (2, N = 852) = 12.331, p = 0.002).

No one reported having used illicit drugs during the past 48 hours, but nearly 2% reported having used illicit drugs during the past 12 months. No one reported being absent from work because of using such drugs during the past 12 months, and no one reported in-efficiency at work because of illicit drug use during the past 12 months. The prevalence of illicit drug use during the past 12 months was significantly higher among young than among older employees (χ^2^ (1, N = 248) = 12.524, p = 0.000) (Table 3).

Self-reported medicinal drug use during the past 48 hours was 4.6%, and 16.7% of these recent users reported that they had been absent from work because of using medicinal drugs the past 12 months. Moreover, 26.2% of the recent users reported in-efficiency at work because of medicinal drug use the past 12 months. The prevalence of medicinal drug use during the past 48 hours was significantly higher among employees aged above 40 years than among younger employees (χ^2^ (1, N = 892) = 11.931, p = 0.001), it was higher among hospital workers than among pharmacy workers (χ^2^ (1, N = 905) = 4.504, p = 0.034) and it was higher among respondents having primary and secondary school as their highest education level than among those with a higher level of education (χ^2^ (2, N = 843) = 7.158, p = 0.028).

When combining results from the questionnaire with analytical results from OF, supplementary information on the recent use of psychoactive substances may be revealed. Detection of drugs in samples of OF indicates, in general, drug use during the past 24–48 hours [23]. The prevalence of self-reported use of illicit drugs was 0%, but the prevalence of positive OF samples was 0.6% suggesting under-reporting of illicit drug use. For alcohol, a lower prevalence of EtG was found in OF than self-reported consumption of four drinks or more the past 24 hours (0.3% versus 0.8%). Under-reporting did not seem to be significant for medicinal drugs either, with a prevalence of 4.6% for self-reported use the past 48 hours versus 3.1% positive samples for medicinal drugs. Under-reporting was less frequent in this study than in the pilot study, suggesting that HPs under-report to a smaller extent than other workers [11]. This information may be useful for future studies.

## Discussion

This study represents the first attempt to estimate the prevalence of alcohol and drug use and impairment and absence due to such use among HPs in Norway and whether such use depends on socio-demographic attributes such as gender, age, educational level and workplace.

### Prevalence of alcohol and drug use measured by questionnaire and oral fluid analysis

The results from this study showed that this sample of HPs seldom used illicit drugs, few had a high level of alcohol consumption, and few tested positive for medicinal drugs. Alcohol and illicit drug use was lower than found in a previous pilot study of employees [11], which did not include HPs. Our findings in OF samples and from questionnaires suggest that the prevalence of problems is less frequent among HPs in Norway than in some other countries. In a survey conducted among student nurses from the US, 11.5% reported past misuse of medications [24]. A study conducted by Kenna and Wood measured the prevalence of alcohol and illicit drug use among dentists and some physicians, revealing a higher prevalence than found in our study [25]. Marijuana was used the past year by 8.8% of the dentists and 3.8% of the physicians. In our study the prevalence was 1.9% for use of all illicit drugs the past year in the sample as a whole. THC has a relatively short detection time in OF and that may be an explanation of why cannabis was not the most frequently detected illicit drug in this study [23,26]. Nevertheless, our findings correspond quite well with the findings of Nesvåg and Lie, were 2.6% reported use of cannabis and/or other illicit drugs once or more during the past 12 months [13].

Psychoactive drugs may cause several problems when used or abused at work, especially if their effects are sedating. Only 4.6% of the participants reported having used such drugs within 48 hours, and most of the use is probably therapeutically use of sleeping agents and tranquilizers.

EtG was found in a very small proportion of the OF samples. A previous study found that an intake of about one bottle of wine gave a positive EtG up to 12 hours after starting drinking [27]. Therefore, a large intake of alcohol is needed to produce sufficient EtG for detection in samples of OF the next day. The variation was large, so the correlation between recent alcohol intake and concentration of EtG in OF is fairly low [27]. This may explain why we did not find more than three positive samples for EtG even though seven respondents reported drinking four or more drinks during the past 24 hours; only one of them had positive EtG. The two other positive EtG samples were found in persons that reported lower alcohol consumption the day before than four or more drinks. The fact that we did not detect any alcohol positive samples implies that none of the respondents had a recent intake of alcohol exceeding a blood alcohol concentration of 0.1 g/L or more at the time of sample collection. Therefore, working under the influence of alcohol did not seem to be a significant problem among this group of HPs; neither was working after a large alcohol intake the day before.

These results may suggest that HPs are more reluctant to use alcohol or illicit drugs than employees in other types of businesses. This assumption is supported by the results from an American study, where employees in the Healthcare and Social Assistance reported less past month illicit drug use and past month heavy alcohol use than most other industries [2].

The use of medicinal drugs was found to be higher, at least partly because of differences in age and gender between this study and the pilot study [11]. In this study, more than 8/10 were women and the age distribution was even, while in the pilot more than 2/3 were men and the age was lower.

### Self-reported absence from and impairment at work due to alcohol and drug use

Self-reported data covering the past 12 months indicated that hangover after alcohol use (12.2%) and absence from work due to drinking alcohol (0.9%) was a larger problem than impairment by alcohol (0%) or drugs at work. Psychoactive drugs may cause several problems when used or abused at work, especially if their effects are sedating. Only 4.6% of the participants reported having used such drugs within 48 hours, and most of the use is probably therapeutically use of sleeping agents and tranquilizers. However, 1/6 of these recent self-reported users reported that they had been absent from work because of use of medicinal drugs during the past 12 months, and more than 1/4 of them reported in-efficiency at work because of the use of medicinal drugs during the past 12 months. The total prevalence of these negative side effects of medicinal drug use was low in this study population. Self-reported in-efficiency or hangover at work due to alcohol and drug use was also significantly less prevalent than found in the pilot study [11], which is the most comparable study that has been performed with both OF samples and questionnaires.

### Alcohol and drug use in relation to age, gender, educational level and workplace

This study did not find any significant gender differences regarding alcohol and drug use. As expected [4,28], the younger employees had a higher alcohol consumption than the older employees, but medicinal drug use was more frequent among older employees than among younger employees. Similar results have been reported by Skretting *et al.*, Gjerde *et al.* and Nesvåg and Lie [13,15,16]. Self-reported in-efficiency or hangover at work due to alcohol during the past 12 months was more frequent among the age group below 40 years and among employees with higher educational level, and the results were consistent with data published by Storvoll *et al.*[17]. Lower educational level and working at a hospital rather than at a hospital pharmacy was associated with more medicinal drug use in this study.

### Strengths/limitations

The strengths of this study were that the participating and testing was done unannounced, that the participation rate was close to 100%, and that all the samples were tested at the same laboratory giving a high analytical reproducibility. A large proportion of the employees (97%) provided samples of OF. In addition, the participation was not time consuming, taking only five minutes. The analytical methods detected a wide range of psychoactive substances. The study was anonymous. Finally, the study included HPs with many different backgrounds, as described in the methods section, providing a wide variety of HPs. One limitation of this study was that a small proportion of the participants did not give information concerning gender, age group, or educational level. Moreover, the included hospitals were of small to middle size, and hospitals and pharmacies were located in the southern part of the country only, and some sectors in the health care business were not included (dentistry, primary health care, private clinics, different public institutions, etc.). Thus, in order to obtain more representative figures of the prevalence of alcohol and drug use among HPs in Norway, future studies would most likely benefit from including HPs from all parts of the country and from a wider range of health care businesses.

Samples of OF only reflect a relatively short time frame; it may be considered as a real-time test: if a drug is detected in a sample of OF it is also likely to be present in blood. Samples of OF have a detection window varying from about 6 h to 48 h after use depending on drug type and dose taken [29]. A long detection time may confuse the interpretation of the results, with shorter detection time, the risk of actually being under the influence of alcohol and drugs is quite high with a concomitant positive sample of OF. This study aimed at trying to measure as recent drug intake as possible, rather than drug intake that no longer has an influence on the work-situation. However, a large intake or several intakes over a few days of a slowly eliminated drug may be detected for a longer time. It is unlikely that such use was common among the drug positive employees.

#### Methodological considerations

None of the OF samples used in this study was collected during the weekends, and approximately 70% of the drinking situations in Norway take place during the weekend [28], it is therefore not very surprising that the prevalence of recent alcohol use, measured as alcohol and EtG in OF, was fairly low in this study. It is also known that illicit drug use is more frequent in the late night during weekends [30]. For that reason, it is reasonable to assume that most of the illicit drug use among this group occurs during weekends (i.e., recreational drug use). When it comes to use of medicinal drugs, they are probably mostly used therapeutically and thus this use is likely to be more evenly distributed through week days and gives a higher prevalence than was the case for illicit drugs and alcohol. Moreover, when using self-reported information it is important to acknowledge the fact that under-reporting is common [31,32] and it is reasonable to expect it to be even more likely for information concerning illicit actions such as illicit drug use as indicated by the findings of the current study and the study by Gjerde *et al.*[11]. A final issue to the methodological considerations is that when analyzing OF, very low concentrations (i.e., below the analytical LLOQ) will not be considered as positive findings. For benzodiazepines the concentrations in OF are mostly much lower compared to blood samples [33]. Finally, analytes that are not included in the analytical repertoire will not be detected nor considered as positive. One major drawback with the self-reported data is the ability to under- and over-report, giving bias to the results. We have tried to reduce this problem by using OF samples.

## Conclusion

The total number of positive samples and self-reported use of alcohol and drugs was low among this sample of HPs; the prevalence of reported and detected drug or alcohol use was lower than in other business sectors in Norway and other countries. Even though intoxication has been reported to be the most common cause of loss of authorization to work in healthcare in Norway, being unfit to work due to acute alcohol or drug intoxication did not seem to be widespread in this group. Overall, few participants reported use of illicit drugs, or having a large consumption of alcohol. Most of the use of medicinal drugs was probably therapeutically use of sleeping agents and tranquilizers. The majority of samples found positive for medicinal drugs were from participants above 40 years old, working at a hospital rather than in a pharmacy, and without education above secondary school. Nevertheless, it should be emphasized that a relatively large fraction of the HPs reported absence from work and reduced efficiency at work due to alcohol and particularly medical drug use during the past 12 months., i.e., impairment due to medicinal drugs and alcohol use appeared to be a bigger issue in the work setting than the effects of acute intoxication or the use of illicit drugs. However, the overall problem was low-prevalent. This study did not include all health care sectors from every part in Norway; therefore future studies would most likely obtain more representative measures of the prevalence of alcohol and drug use among all HPs by including HPs from all parts of the country and from a wider range of health care businesses.

## Abbreviations

6-AM: 6-Acetylmorphine; 7-AC: 7-Aminoclonazepam; 7-AF: 7-Aminoflunitrazepam; 7-AN: 7-Aminonitrazepam; EtG: Ethyl glucuronide; HP: Health professional; LLOQ: Lower limit of quantification; MDMA: 3,4-Methylenedioxymethamphetamine; NIPH: Norwegian Institute of Public Health; OF: Oral fluid; THC: ∆9-tetrahydrocannabinol; WDT: Workplace drug testing.

## Competing interests

The authors declare that they have no competing interests.

## Authors’ contributions

All authors participated in designing the study and interpreting the data. HMEE had the main responsibility for planning and coordinating the acquisition of data and had the main responsibility for drafting the manuscript. All co-authors contributed in revising the manuscript critically for intellectual content. All authors read and approved the manuscript.
